# Clinical gerontological nursing competence among licensed practical nurses in healthcare services—A descriptive cross‐sectional study

**DOI:** 10.1111/scs.13312

**Published:** 2024-11-18

**Authors:** Suonnansalo Petra, Pramila‐Savukoski Sari, Meriläinen Merja, Siira Heidi, Sneck Sami, Tohmola Anniina, Karsikas Eevi, Tuomikoski Anna‐Maria

**Affiliations:** ^1^ The Wellbeing Services County of North Ostrobothnia Oulu University Hospital Oulu Finland; ^2^ Research Unit of Health Sciences and Technology, Faculty of Medicine University of Oulu Oulu Finland; ^3^ Medical Research Center Oulu Oulu University Hospital and University of Oulu Oulu Finland; ^4^ Research Unit of Health Sciences and Technology/GeroNursing Centre, Faculty of Medicine University of Oulu Oulu Finland; ^5^ Lapland University of Applied Sciences Lapland Finland

**Keywords:** competence, cross‐sectional study, gerontological nursing, healthcare, licensed practical nurse

## Abstract

**Aim:**

This study aimed to describe self‐assessed clinical gerontological nursing competence and its associated factors among licensed practical nurses.

**Design:**

A descriptive cross‐sectional design was adopted for the study.

**Methods:**

Data were collected in Autumn 2023 from 394 licensed practical nurses working in healthcare services for older people in one well‐being services county in Finland. The nurses, recruited through convenience sampling, were surveyed using a 40‐item self‐assessment clinical gerontological nursing competence instrument (on a Likert scale from 1 = poor to 5 = excellent). Descriptive statistical methods were used to analyse the results.

**Results:**

Participants mainly assessed their clinical gerontological competence as good. Competence in using assistive devices to support functional ability was assessed as very good, while competence in postoperative wound care was assessed as the weakest. Participants working in 24‐h services assessed their competence as the weakest among the three clinical gerontological nursing areas. Participants under 40 years of age with 5–10 years of work experience self‐assessed statistically significantly stronger competence in disease‐specific nursing than those over 40 with less work experience.

**Conclusion:**

The self‐assessed competence of licensed practical nurses varies across different service and care units. Competence in wound care requires more focus and education in the future. Attention should also be paid to competence development in different educational areas, for example, through continuous education and competence development models. The results could facilitate licensed practical nurses' competence development and management in clinical gerontological nursing. The study's insights can also guide allocating resources and education to ensure high‐quality care in different service areas.

## INTRODUCTION

Increasing life expectancy is leading to a rise in the number of older people worldwide. The number of people aged 80 and above is expected to triple by 2050, reaching 426 million [1]. According to WHO [World Health Organisation], the ageing of the global population poses a challenge for healthcare systems [2]. It is expected to increase disabilities and deaths from noncommunicable diseases, also known as chronic diseases, worldwide [2]. Healthcare professionals play a major role in promoting the health and functional ability of older people, and licensed practical nurses (LPNs) play the largest role alongside registered nurses (RNs) [3]. LPNs focus more on basic patient care, while RNs have greater responsibility, for example, for patient's medication. However, in earlier studies, it has been discussed that LPNs' role in clinical practice may have expanded to the tasks more closely belonging to a RN, especially when there are no RNs available [4, 5]. The role of LPSs can be unclear and they are faced with different educational expectations [6].

The role of LPNs is emphasised in healthcare, but studies on their profession and competence development are scarce [6, 7]. Although the growing needs of older people care have been recognised [1] and gerontological competence in nursing has been identified [8, 9], there is a distinct gap in research on the clinical gerontological competence of LPNs. Clinical competence refers to the knowledge, skills and attitudes associated with practice‐based nursing [10]. As LPNs are the closest professionals to older patients, their competence plays an important role in older peoples' daily lives and in providing quality care [4] and dignified ageing [3]. It is crucial to understand LPNs' self‐assessments and the factors influencing their clinical gerontological competences to allocate resources correctly, avoid inappropriate investigations, and develop continuous learning and competence management. Moreover, it makes economic sense to develop care and high‐quality services for older people [2]. Continuous professional turnover challenges competence development and orientations as well as evidence‐based practices and staff development [8]. With the global shortage of nursing staff, research on LPNs is needed to boost the attractiveness of the profession [7].

## BACKGROUND

Definitions of older people vary. Generally, the phrase is used to refer to people 60 years of age or older [1]. In the Finnish context, the term ´older people´ refers to people aged 65 or older based on their entitlement to an old‐age pension [11]. Ageing causes a decline in physical, social and psychological ability, increasing, for example, changes in the immune system and the risk of long‐term illnesses and multimorbidity [1, 2]. Age‐related functional changes and diseases require special attention and pose additional healthcare needs. Older people are at greater risk of contracting infections and being prescribed medications that require monitoring [12]. Impairment of physical capacity through loss of muscle strength increases the risk of falls [13]. The risk of falls is further increased by medications for various diseases [14]. Due to the changes in functional capacity, the incidence of pressure ulcers may increase among older people and preventing those requires competence [15]. Chronic wounds may challenge the care [16]. Older people suffer from hearing and/or memory loss or need support in rehabilitation [17]. All in all, older people's different service needs lead them to live in different types of living arrangements, either in their own homes, or for example, in care homes where the services are provided [3, 18]. Severe needs require a massive amount of social and healthcare resources and create a high demand for competence in clinical gerontological nursing, for example for LPNs, to guarantee active and functional ageing [2, 19].

The form of the LPN profession and associated education varies worldwide. In England, nursing associates earn a special two‐year degree and work across all fields of nursing (adults and children) [20]. In the United States, LPNs work in primary care, long‐term care, and home care services, providing practical nursing care [21]. The role of LPNs in providing high‐quality care has expanded, especially in Finland [7], where they form the second most popular profession, with around 80,000 LPNs in 2020 [22]. In Finland, an LPN has an upper secondary school degree that can be completed in a vocational institution. The education consists of 180 ECTs (The European Credit Transfer and Accumulation System) and a student may choose from different areas of specialisation, such as rehabilitation for older people or paediatric care. Generally, LPNs competences include older people's care, foot, home and restorative care [23]. In addition, they are competent in promoting health [23]. LPNs can register as both social and healthcare professionals [24] and it is a protected occupational title. While RNs must undergo an extensive medication management education to satisfy their qualifications, for LPNs this education is delivered at a basic level but can be extended with additional qualifications [24]. At its widest extent, the job description of an LPN might further expand and need a deeper understanding [4].

LPNs' competence in older people's care is essential since they work close to them [7, 8]. Generally, competence can be thought of as a confluence of knowledge, skills, attitudes and performance that enables efficient action [25]. Gerontological nursing requires a broader set of competences than basic nursing care [26], including attitudinal, ethical, interactional, evidence‐based care, pedagogical, leadership and development competences [17]. More specifically, gerontological nursing requires clinical competence, including, for example, knowledge of psychosocial and biological ageing changes and geriatric diseases, competence in health and well‐being promotion, interaction and ethics [9, 27, 28]. Although, gerontological nursing competence has been rated as a good level in respectful encounters [28] and knowledge of essential clinical practice [26], it needs to be developed [29] in areas such as wound care [30, 31] and support for older people's sexuality [28]. Rababa et al. [32] have found that the professional characteristics of nurses have an impact on their knowledge and attitudes toward caring for older people [32]. Generally, previous studies regarding gerontological nursing focus mainly on RNs and based on our knowledge, not that much specifically clinical gerontological competence. This study focuses on the clinical competence of nursing professionals, like competence in disease‐specific nursing/care, pressure ulcer prevention and devices supporting functional ability.

Understanding the LPNs' self‐assessed competence has an impact nationally and globally. In Finland, 21 self‐governing, government‐funded regions, ‘well‐being services counties’, are responsible for organising equal and economically sustainable health, social and emergency services [33]. Developing professionals' competence and equitable gerontological care is important in well‐being services counties [3]. Adequate, high‐quality clinical gerontological nursing competence ensures the quality of care and patient safety [8] in addition to the well‐being and commitment of the staff [19]. Clinical gerontological nursing competence also enables LPNs to provide holistic, individualised care [8] and support the needs of the older people [4, 17]. A shortage of staff and remote work areas pose challenges to the daily work of LPNs [5]. LPNs role is perceived as subordinate and competences should be considered more carefully at the organisational level [4] and their professional identity and continuous education should be strengthened accordingly [4, 6].

Although researchers have enquired into which gerontological competences nursing professionals need in general and what to develop, there is no clear understanding of the existing clinical gerontological competences LPNs demonstrate in various care units and background variables that are associated with that competence. This study aims to fill a global knowledge gap regarding LPNs' clinical gerontological competence evaluated by themselves. This research will help develop the structures necessary for developing the competences of LPNs at different stages of their careers and different services for older people. Prevention, early diagnosis and effective treatment can help support older people's health and improve their quality of life [8]. To improve the field's attractiveness and retention in the social and health sectors, their work and competence should be highlighted and strengthened [34].

## THE STUDY

### Aim and research questions

This study aimed to describe LPNs' self‐assessed competence in clinical gerontological nursing and its associated factors. The research questions were as follows:

1. What kind of self‐assessed clinical gerontological nursing competence levels do LPNs have in healthcare services?
2. What background variables are associated with LPNs' self‐assessed clinical gerontological nursing competence levels in healthcare services?

## METHODS

### Study design

This study adopted a cross‐sectional design [35]. The Strengthening the Reporting of Observational Studies in Epidemiology (STROBE) checklist was used in reporting this study [36].

### Participants

The data were collected from LPNs (*n* = 394) working in geriatric care units in one well‐being services county (with around 400,000 residents) in Finland. The convenience sampling method described by Polit and Beck [35] was used to select the participants. The inclusion criteria required that each participant had to (1) work as an LPN in one well‐being services county in Finland, (2) be currently working in older people care and (3) work either in 24‐h services, an assessment and rehabilitation unit, or home care services for older people. LPNs working in remote home care, private service units, service management, or security alarm centres were excluded from the study due to the aim and focus of the project.

### Instrument

The data reported in this study were collected using a clinical gerontological nursing competence self‐assessment instrument. The items (*n* = 40) were developed to address essential competence areas in clinical gerontological nursing and based on research [8, 28], evidence about older people care [37] and the expertise of gerontological and nursing science experts. Items were evaluated by gerontological and nursing sciences experts (*n* = 8) regarding the relevance and clarity of the items (I‐CVI, content validity index) and the whole entity of 40 items (S‐CVI/Ave). The limit for an acceptable I‐CVI score was set as ≥0.78 for items and for the whole instrument, S‐CVI/Ave was set to be 0.80–1.00 [38]. Both scores were evaluated as satisfying levels (≥0.80) and only small modifications were made.

The self‐assessment instrument included 21 background questions and 40 items on clinical gerontological competence. The background questions collected data on each participant's age, working unit, work experience in social services and healthcare, work experience in older people care, education (highest completed degree), participation in professional events, expert networks, internal and external training in the organisation, following of professional social media and experience working as an educator within the previous 2 years. Information about participation in continuing education and different types of education was also solicited.

The 40 items related to clinical gerontological competence were reported on a five‐point Likert scale (*1* = *poor*, *2* = *fair*, *3* = *good*, *4* = *very good*, *5* = *excellent*). The reliability of the instrument was tested with Cronbach's alpha, with scores of 0.984 for factor 1, *competence in disease‐specific nursing* (23 items); 0.962 for factor 2, *competence in wound care and pressure ulcer prevention* (11 items); 0.804 for factor 3, *competence in using assistive devices to support functional ability* (4 items); and 0.888 for factor 4, *competence in postoperative wound care* (2 items); (see Table 1). The overall reliability of the instrument was 0.982.

**TABLE 1:** The reliability of the instrument.

Factors describing LPNs' self‐assessed competence in clinical gerontological nursing	Factor items measured on a five‐point Likert scale (1 = poor, 2 = fair, 3 = good, 4 = very good, 5 = excellent)	Factor 1	Factor 2	Factor 3	Factor 4
Competence in disease‐specific nursing	I can assess the care needs of an older person suffering from sudden disorientation	0.841			
	2 I can assess the care needs of an older person suffering from dizziness	0.833			
	3 I can assess the care needs of an older person with a headache	0.821			
	4 I can assess the need for treatment of an older person with abdominal pain	0.812			
	5 I can assess the care needs of an older person with respiratory dyspnoea	0.808			
	6 I can assess the care needs of an older person with a decrease in general condition	0.803			
	7 I can assess the care needs of an older person with chest pain	0.783			
	8 I can assess the care needs of an older person who has fallen	0.776			
	9 I can care for an older person with heart failure	0.769			
	10 I can treat an older person with coronary heart disease	0.764			
	11 I can care for an older person with functional deficits due to a circulatory problem in the brain	0.753			
	12 I can care for an older person with chronic pain	0.746			
	13 I can treat older people with respiratory and lung diseases (e.g., asthma, COPD)	0.744			
	14 I can care for an older person with Parkinson's disease	0.736			
	15 I can treat older people with musculoskeletal disorders (e.g., osteoarthritis, rheumatoid arthritis, osteoporosis)	0.716			
	16 I can deal with medical emergencies	0.690			
	17 I can care for an older person with cancer	0.684			
	18 I can care for an older person with eye disease	0.680			
	19 I can care for older people with diabetes (type 1 and type 2)	0.674			
	20 I can deal with unexpected death situations (the person is found in his/her environment)	0.652			
	21 I can care for a person with urinary incontinence	0.631			
	22 I can care for an elderly person with neurasthenia	0.624			
	23 I can resuscitate a person	0.597			
Competence in wound care and pressure ulcer prevention	24 I can treat wounds with different wound dressings		0.811		
	25 I can assess wound bleeding and exudation		0.808		
	26 I can assess the condition of the wound		0.805		
	27 I can clean a wound		0.778		
	28 I can explain the general principles of wound care (e.g., different types of wounds, wound care products)		0.756		
	29 I can care for a wound aseptically		0.733		
	30 I can accurately document wound care in client and patient records		0.719		

	31 I can assess the need for pain management before and after wound care		0.714		
	32 I can use different devices to care for a wound		0.620		
	33 I can assess the risk of pressure ulcers		0.606		
34 I can carry out foot care (including the specifics of diabetic foot care)		0.558		
Competence in using assistive devices to support functional ability	35 I can put on support ties/socks			0.675	
	36 I can maintain a hearing aid			0.598	
	37 I can care for stoma and stoma‐related skin care			0.598	
	38 I can put on and maintain a prosthesis			0.553	
Competence in postoperative wound care	39 I can aseptically remove skin barrier clips				0.799
	40 I can remove sutures aseptically				0.774
Eigenvalue		25.214	2.552	1.459	1.207
Percentage of variance explained					63.036
Total percentage of variance explained by the factor model					76.080
Cronbach's alpha		0.984	0.962	0.804	0.888
Cronbach's alpha for total scale					0.982

*Note*: Extraction method: Principal component analysis. Rotation method: Varimax with Kaiser normalisation rotation converged in 7 iterations.

### Data collection

The data were collected as part of a larger Finnish project called *Age is Cool*. Other portions of the data collected in the project, gathered using several different instruments (like the GeroNursingCom instrument), have been reported in separate publications [39]. The data were collected electronically using a Webropol® 3.0 questionnaire between June and August 2023. A link to the questionnaire was sent to the contact persons of the units in one of the well‐being services counties in Finland, who forwarded it via email to the LPNs and RNs they employed (*n* = 3112). The sample size was calculated and the goal was to get at least five respondents per item (at least 300 respondents) [40]. The goal was reached (*n* = 394). The data were collected over a 12‐week period, during which two email reminders were sent. This study reports the results of the LPNs' responses.

### Data analysis

The data were analysed using IBM SPSS Statistics 27. The instrument (40 items) was not validated, so an exploratory factor analysis (EFA) was performed. An EFA identifies hypothetical constructs to explain the covariation among a set of measured variables [35]. Five‐factor models were applied, and finally, a model for four‐sum variables was statistically and theoretically constructed. Kaiser–Meyer–Olkin's measure of sampling adequacy was 0.977 and Bartlett's test of sphericity value (x^2^ = 19287.829, df = 780) was acceptable (*p* < 0.005). Competence is presented in terms of mean, median and range of competence. Competence was divided into three levels (fair = 1–2.499, good = 2.5–3.499 and very good = 3.5–5). This classification scheme has been used before in the analysis of gerontological nursing competence [28], which makes it easier to compare our results with past findings. The distribution of LPNs across the different areas of competence is presented using percentages and frequencies.

Descriptive statistical methods were used to analyse the background variables. The limit for statistical significance was set at a *p*‐value <0.05 [35]. One‐way analysis of variance (ANOVA) and the independent samples *t*‐test were used to test the relationships between background variables in different groups. The results were confirmed by the Benjamini‐Hochberg procedure (False Discovery Rate) procedure. It is a statistical method used in multiple hypothesis testing to control the expected proportion of false discoveries. Generally, there were no missing data due to the mandatory response function in Webropol.

### Ethical considerations

This study followed the ethical principles for medical research set out in the Helsinki Declaration [41]. Permission for the research with ethical approval was granted from the board of the well‐being services county on 01/03/2023. Research ethics committee approval was not required since the study did not involve minors, clinical trials, or direct or indirect physical or physiological harm to the participants [42, 43]. An electronic invitation was sent to the participants, explaining the purpose of the study, its objective, assurances about anonymity, confidentiality, and data handling and a reminder of the right to withdraw from the study without consequences. Informed consent was collected from each participant in accordance with the EU's General Data Protection Regulations (GDPR) [44]. The research data have been kept confidential as required by the GDPR [44]. It will be destroyed after 10 years, as planned in the project's data management plan and common guidelines in Finland.

## RESULTS

### Characteristics of the participants

The results of the study indicate that more than 80% of participants worked either in home care or 24‐h services. Only 7.6% of the participants worked in an assessment and rehabilitation unit. Almost half of the participants were over 50 years old. The younger age groups were almost evenly split between those under 40 (30.5%) and those aged 40–49 (22.3%). Similarly, work experience in social and healthcare settings was almost evenly split between less than 10 years and over 20 years. Of the participants, 37.8% reported working in services for older people for more than 15 years, and around half of the respondents had between 5 and 15 years of experience. For almost all participants, the highest level of education completed was a vocational degree.

### LPNs' self‐assessed competence in clinical gerontological nursing

The LPNs assessed their clinical gerontological nursing competence as good, with a mean of 3.346 (SD = 0.699) (see Table 2). The strongest competence was reported in the area of using assistive devices to support functional ability, where it was assessed as very good, with a mean of 3.561 (SD = 0.824). Competence in disease‐specific nursing, wound care and pressure ulcer prevention, and competence in postoperative wound care were assessed as good, with means ranging between 2.520 and 3.453. Although, most of the participants rated their competence as good or very good in these competence areas, half of the participants rated their competence in postoperative wound care as fair.

**TABLE 2:** LPNs' self‐assessed competence levels in a dimension of clinical gerontological nursing.

Dimension in clinical gerontological competence (*n* = 394)	Number of items	Mean (SD)	Median (range)	Fair^a^ *n* (%)	Good^b^ *n* (%)	Very good^c^ *n* (%)
Competence in disease‐specific nursing	23	3.453 (0.711)	3.434 (3.22)	28 (7.1)	182 (46.2)	184 (46.7)
Competence in wound care and pressure ulcer prevention	11	3.193 (0.798)	3.045 (4.00)	76 (19.3)	167 (42.4)	151 (38.3)
Competence in using assistive devices supporting functional ability	4	3.561 (0.824)	3.500 (3.25)	30 (7.6)	145 (36.8)	219 (55.6)
Competence in postoperative wound care	2	2.520 (1.272)	2.250 (4.00)	197 (50.0)	91 (23.1)	106 (26.9)
Clinical gerontological nursing total competence	40	3.346 (0.699)	3.325 (3.325)	39 (9.9)	179 (45.4)	176 (44.7)

*Source*: Authors' own work.

Abbreviation: SD, standard deviation.

^a^ Fair = 1–2.499.

^b^ Good = 2.5–3.499.

^c^ Very good = 3.5–5.

### Background variables' effects on LPNs' self‐assessed competence in clinical gerontological nursing

Among the LPNs, the work unit had a statistically significant association with self‐assessment in *competence in disease‐specific nursing* (*p* = 0.005), *wound care and pressure ulcer prevention* (*p* = 0.049), and *postoperative wound care* (*p* < 0.001; see Table 3). The LPNs working in assessment and rehabilitation units reported the highest competences in *disease‐specific nursing*, *wound care and pressure ulcer prevention*, and *postoperative wound care*. The participants working in 24‐h services self‐assessed the lowest levels in three of four competence areas with the lowest rate in postoperative wound care (mean = 2.536, SD = 1.216). Age had a statistically significant association with three rated competence areas. Participants under the age of 40 reported statistically significantly stronger competence in *disease‐specific nursing*, *wound care and pressure ulcer prevention*, and *using assistive devices to support functional ability* compared to other age groups. Participants over 50 years of age reported the lowest levels of competence in all four areas.

**TABLE 3:** The association between the background variables and LPNs' self–assessed competence levels in clinical gerontological nursing (*n* = 394).

Background variable	Total sample (*n* = 394)	F1 Competence in disease–specific nursing (mean, SD)	*p*	F2 Competence in wound care and pressure ulcer prevention (mean, SD)	*p*	F3 Competence in using assistive devices to support functional ability(mean, SD)	*p*	F4 Competence in postoperative wound care (mean, SD)	*p*
Working unit			**0.005** ^a^		**0.049** ^a^		0.113^a^		**<0.001** ^a^
Home care services	182 (46.2)	3.426 (0.706)		3.173 (0.855)		3.596 (0.839)		2.373 (1.314)	
Assessment and rehabilitation	30 (7.6)	3.717 (0.706)		3.324 (0.731)		3.608 (0.687)		3.066 (1.006)	
24–h services	179 (45.4)	3.418 (0.700)		3.172 (0.737)		3.501 (0.825)		2.536 (1.216)	
Not reported	3 (0.8)	4.594 (0.559)		4.393 (0.555)		4.583 (0.520)		5.000 (0.000)	
Age group, years			**0.021** ^a^		**0.003** ^a^		**0.036** ^a^		0.225^a^
Under 40	120 (30.5)	3.589 (0.710)		3.391 (0.795)		3.714 (0.827)		2.620 (1.330)	
40–49	88 (22.3)	3.467 (0.666)		3.176 (0.762)		3.554 (0.764)		2.630 (1.271)	
Over 50	186 (47.2)	3.359 (0.722)		3.074 (0.795)		3.466 (0.840)		2.403 (1.231)	
Work experience in healthcare settings, years			0.605^a^		0.884^a^		0.471^a^		0.817^a^
Under 10	142 (36.0)	3.479 (0.729)		3.180 (0.827)		3.537 (0.843)		2.485 (1.265)	
10–19	126 (32.0)	3.401 (0.637)		3.179 (0.754)		3.515 (0.775)		2.500 (1.237)	
Over 20	126 (32.0)	3.477 (0.763)		3.222 (0.813)		3.634 (0.853)		2.579 (1.322)	
Work experience in older people care, years			**0.044** ^a^		0.849^a^		0.065^a^		0.479^a^
Under 5	66 (16.8)	3.330 (0.709)		3.133 (0.773)		3.393 (0.850)		2.348 (1.240)	
5–10	92 (23.4)	3.618 (0.694)		3.188 (0.844)		3.698 (0.791)		2.614 (1.324)	
10–15	87 (22.1)	3.371 (0.605)		3.175 (0.734)		3.456 (0.765)		2.436 (1.188)	
Over 15	149 (37.8)	3.455 (0.766)		3.234 (0.820)		3.612 (0.854)		2.587 (1.304)	
Highest completed degree			**0.001** ^a^		**0.005** ^a^		**0.015** ^a^		**0.010** ^a^
Vocational degree	378 (95.9)	3.459 (0.700)		3.199 (0.796)		3.562 (0.815)		2.517 (1.270)	
Bachelor's degree (university or a university of applied sciences)	4 (1.0)	4.423 (0.525)		4.159 (0.571)		4.562 (0.718)		4.250 (0.957)	
Master's degree (university or a university of applied sciences), doctoral degree, and others	12 (3.0)	2.967 (0.770)		2.697 (0.585)		3.187 (0.936)		2.041 (1.010)	
Participation in professional events (e.g., scientific conferences and National Wound Days) in the last 2 years			0.830^b^		0.267^b^		0.867^b^		0.710^b^
Yes	47 (11.9)	3.432 (0.745)		3.315 (0.817)		3.542 (0.869)		2.5851 (1.311)	
No	347 (88.1)	3.456 (0.708)		3.177 (0.795)		3.564 (0.819)		2.511 (1.269)	
Participation in expert networks (e.g., an organisation's own internal, national, or international network) in the last 2 years			0.359^b^		0.866^b^		0.954^b^		0.210^b^
Yes	54 (13.7)	3.371 (0.795)		3.176 (0.875)		3.555 (0.765)		2.722 (1.250)	
No	340 (86.3)	3.467 (0.698)		3.196 (0.786)		3.562 (0.835)		2.488 (1.275)	
Participation in the organisation's internal/external education in the last 2 years			0.218^b^		0.248^b^		0.397^b^		0.533^b^
Yes	303 (76.9)	3.478 (0.698)		3.219 (0.792)		3.580 (0.804)		2.498 (1.285)	
No	91 (23.1)	3.373 (0.752)		3.108 (0.816)		3.497 (0.890)		2.593 (1.233)	
Following professional social media in the last 2 years			0.051^b^		**0.023** ^b^		0.092^b^		0.340^b^
Yes	119 (30.2)	3.560 (0.688)		3.333 (0.759)		3.668 (0.781)		2.613 (1.242)	
No	275 (69.8)	3.407 (0.718)		3.133 (0.808)		3.515 (0.840)		2.480 (1.286)	
Working as an educator in the last 2 years			**0.001** ^b^, ^c^		0.277^b^		0.953^b^		0.443^b^
Yes	6 (1.5)	3.753 (1.221)		3.545 (1.078)		3.541 (1.041)		2.916 (1.744)	
No	388 (98.5)	3.449 (0.702)		3.188 (0.793)		3.561 (0.822)		2.514 (1.266)	
Completing one continuous education courses			0.141^b^		**0.019** ^b^		0.152^b^		0.256^b^
Yes	58 (14.7)	3.326 (0.744)		2.967 (0.876)		3.418 (0.920)		2.344 (1.301)	
No	336 (85.3)	3.475 (0.705)		3.233 (0.778)		3.586 (0.806)		2.550 (1.267)	
Completing 2–5 continuous education courses			0.881^b^		0.273^b^		0.806^b^		0.835^b^
Yes	243 (61.7)	3.449 (0.713)		3.159 (0.779)		3.553 (0.835)		2.530 (1.284)	
No	151 (38.3)	3.460 (0.711)		3.249 (0.826)		3.574 (0.809)		2.503 (1.257)	
Completing over 5 continuous education courses			0.162^b^		**0.001** ^b^		**0.043** ^b^, ^c^		0.479^b^
Yes	93 (23.6)	3.544 (0.681)		3.426 (0.745)		3.672 (0.720)		2.602 (1.225)	
No	301 (76.4)	3.426 (0.720)		3.122 (0.801)		3.527 (0.852)		2.495 (1.288)	
Completing education regarding wound care			0.578^b^		**< 0.001** ^b^		**0.010** ^b^, ^c^		0.679^b^
Yes	128 (32.5)	3.482 (0.693)		3.415 (0.777)		3.640 (0.730)		2.558 (1.228)	
No	266 (67.5)	3.440 (0.721)		3.087 (0.787)		3.523 (0.865)		2.501 (1.295)	
Rehabilitative care education (GeroNursingCentre)			0.801^b^		0.828^b^		0.794^b^		0.948^b^
Yes	52 (13.2)	3.430 (0.738)		3.171 (0.808)		3.533 (0.807)		2.509 (1.234)	
No	342 (86.8)	3.457 (0.708)		3.197 (0.797)		3.565 (0.828)		2.521 (1.280)	
Completing education regarding falls			0.336^b^		0.066^b^		0.298^b^		0.238^b^
Yes	85 (21.6)	3.519 (0.721)		3.334 (0.795)		3.644 (0.783)		2.664 (1.247)	
No	309 (78.4)	3.435 (0.709)		3.155 (0.796)		3.538 (0.835)		2.480 (1.279)	
Completing education regarding predicting infections			0.547^b^		**0.038** ^b^		0.251^b^		0.145^b^
Yes	75 (19.0)	3.498 (0.651)		3.366 (0.724)		3.660 (0.725)		2.713 (1.249)	
No	319 (81.0)	3.443 (0.726)		3.153 (0.810)		3.538 (0.845)		2.474 (1.276)	
Completing education in care needs assessment (NEWS, ISBAR, cABCDE)			**0.011** ^b^		**0.003** ^b^		0.125^b^		**0.049** ^b^
Yes	99 (25.1)	3.611 (0.700)		3.396 (0.814)		3.671 (0.769)		2.737 (1.306)	
No	295 (74.9)	3.400 (0.709)		3.125 (0.782)		3.524 (0.840)		2.447 (1.255)	
Completed RAI (resident assessment instrument) online education			0.528^b^		**0.042** ^b^		0.789^b^		**0.024** ^b^
Yes	156 (39.6)	3.481 (0.690)		3.294 (0.767)		3.575 (0.772)		2.698 (1.256)	
No	238 (60.4)	3.435 (0.726)		3.127 (0.812)		3.552 (0.859)		2.403 (1.272)	
RAISoft online education			0.059^b^		**< 0.001** ^b^		**0.015** ^b^		**0.005** ^b^, ^c^
Yes	247 (62.7)	3.506 (0.717)		3.308 (0.808)		3.639 (0.803)		2.510 (1.335)	
No	147 (37.3)	3.366 (0.696)		3.001 (0.744)		3.430 (0.845)		2.537 (1.164)	
Basic education delivered by a RAI educator			0.109^b^		**0.039** ^b^		0.067^b^		0.846^b^
Yes	108 (27.4)	3.547 (0.688)		3.329 (0.763)		3.685 (0.768)		2.500 (1.266)	
No	286 (72.6)	3.418 (0.718)		3.142 (0.806)		3.514 (0.841)		2.528 (1.277)	

*Note*: *p* = *p*‐value. bolded = statistical significance *p* < 0.05.

Abbreviation: SD, standard deviation.

^a^ One‐way analysis of variance (ANOVA).

^b^ Independent samples *t*–test.

^c^ Exact sig.

Work experience in older people's care was statistically significantly associated with ratings of *competence in disease‐specific nursing* (*p* = 0.044). The highest competence was reported among those who had worked in services for older people for 5–10 years and the weakest among those who had worked in services for older people for under 5 years. The highest completed degree was statistically significantly associated with all competence areas (*p* < 0.05 in all areas). Respondents with a bachelor's degree rated the highest competence in all four areas.

Participation in professional events, expert networks, and internal/external organisational education in the last 2 years was not rated statistically significantly to be associated with competence areas. Following professional social media was statistically significantly associated with the reported *competence in wound care and pressure ulcer prevention* (*p* = 0.023). Those who followed professional social media reported higher competence in this category than those who did not. Having worked as an educator was statistically significantly associated with higher *competence in disease‐specific nursing* than not having done so.

Competence was reported to be stronger in two competence areas if five or more courses had been attended. Turning to the specific continuing education and training courses, the respondents who had completed education on wound care rated statistically significantly stronger *competence in wound care and pressure ulcer prevention* and *using assistive devices to support functional ability* compared to those who had not taken these courses. LPNs who had completed education on infection control reported statistically significantly higher *competence in wound care and pressure ulcer prevention* compared to those who had not. LPNs who had completed education in care needs assessment (NEWS [National Early Warning Score], ISBAR [Identify, Situation, Background, Assessment, Recommendation] and cABCDE [Airway, Breathing, Circulation, Disability, Exposure]) rated statistically significantly stronger competence in three competence areas. Restorative care education and education on falls were not statistically significantly linked to self‐assessed competence in any area.

## DISCUSSION

This study describes LPNs' self‐assessed competence in clinical gerontological nursing and the factors associated with this competence. The responsibility of LPNs to deliver practical care is explicit [4, 7]. In this study, LPNs generally rated their level of competence in clinical gerontological nursing as good. Similar results have been found in earlier studies. In Finland, Kiljunen et al. [29] found that 65% of LPNs and RNs self‐assessed their competence in older people's care as adequate. Faronbi et al. [26] studied RNs' knowledge in older people's care and stated that around 60% demonstrated good knowledge of essential clinical practices in gerontological nursing. Kiljunen et al. [29] found that one‐third of their respondents reported inadequate competence in older people nursing.

In our study, most LPNs assessed their *competence in disease‐specific nursing* as being either very good or good. This may be due to the increasing responsibility and competence required for the basic care of older people [7] and, thus, their competence and diverse knowledge in dealing with older people with a wide range of conditions and non‐communicable diseases [2]. RNs, on the other hand, are generally aware of their professional role in caring for older people, but they may not have enough time to care for older people [45]. This study also found that LPNs reported a high level of *competence in wound care and pressure ulcer prevention*. Still, about one‐fifth of the LPNs reported their competence as fair in that area. Earlier it has been suggested, that LPNs' competence in wound care needs to be further developed [30, 31] which is supported by our findings. One solution could be continuous education, as this study found that participation in continuous education focused on wound care is statistically significant for self‐assessed competence in wound care and pressure ulcer prevention. In particular, the prevention of pressure ulcers should be addressed in healthcare settings to ensure patient safety and the quality of life of older people [15, 46]. In addition, competence in wound care and pressure ulcer prevention among LPNs who followed professional social media was evaluated as stronger than among those who did not, so social media and the evidence‐based information presented here may help to develop competences.

*Competence in using assistive devices to support functional ability* was the strongest self‐assessed area of competence in clinical gerontological nursing. This is an expected result, as LPNs may be the professionals most responsible for the basic care of older people [7] and thus they are familiar with using assistive devices that further might reflect their high assessments of competence. Half of the LPNs rated their c*ompetence in postoperative wound care* as fair. This is true even though it is becoming increasingly challenging to identify the differences between the job descriptions of LPNs and RNs [5]. The expansion of the job description of LPNs may be due to a massive shortage of RNs. Some LPNs even have authorisation to use medical products, while others do not [4]. However, whether the job description of an LPN includes postoperative wound care may vary. In some cases, postoperative wound care is provided by LPNs in a hospital rather than the healthcare services for older people [47]. Thus, postoperative wound care may not be part of the core competence of the LPNs in this study, which may explain the lower level of self‐assessed competence. In any case, about a quarter rated their competence in postoperative wound care as very good. As healthcare services must also consider financial aspects, could LPNs, through competence development and the help of digitalisation, save resources by treating patients instead of the patients being transferred from their home or care facility to an institution to receive postoperative wound care?

Turning to the background variables associated with self‐assessment in clinical gerontological competence, statistically significant associations were found with working unit, age, work experience in older people care, highest completed degree, professional social media usage, whether the LPN had worked as an educator within the last 2 years, and multiple factors on continuous education. In terms of working units, LPNs working in an assessment and rehabilitation unit rated their competence as the strongest in all areas, while those working in 24‐h services rated all areas as the weakest, except for their competence in postoperative wound care. The job description in acute care may be more varied and closer to the work of RNs [5]. Acute care is included in assessment and rehabilitation units. Long‐term care has been identified as challenging for graduate nurses [48]. The question arises of how to develop competence in 24‐h services and how resource levels should be balanced between LPNs and RNs in between different work units/services. For example, in Australia, LPNs have reported that they have a high workload [5].

Our study found that LPNs under 40 years old rated themselves as the most competent of all age groups in different areas, except in postoperative wound care, where they assessed themselves as the least competent. This finding is consistent with former research [29, 49]. In a study by Bing‐Jonsson et al. [49], older nursing staff reported weaker competence in older people's care, while younger staff reported stronger abilities. It is only after graduation that younger LPNs gain up‐to‐date and evidence‐based knowledge to apply in their clinical practice, which may contribute to the results of this study. On the other hand, older professionals may self‐assess their competence critically, which might result in low assessments. They may have broad experience and have noticed what they don't know. Still, continuous education should be provided for LPNs [2], as it has been a predictor of LPNs reported competence before [29]. In our study, statistical significance was evident when an LPN had completed one or more than five continuous education courses. We also found several statistically significant relationships between the continuous education received and the self‐assessed clinical gerontological nursing competence areas. For example, disease‐specific nursing competence was evaluated to have improved if an LPN had worked as an educator within the last 2 years.

LPNs with 5–10 years of experience in older people care reported statistically significantly higher levels of competence in disease‐specific nursing compared to other groups of work experience. This is a similar result to the study by Kiljunen et al. [29], according to which the length of work experience predicted the self‐assessed competence of nursing staff. The respondents, who self‐assessed their competence in all areas as high, had a bachelor's degree as the highest completed degree. In Finland, LPNs have vocational degrees, so the participants in this study may be qualified RNs working as LPNs. However, participants who reported a master's or doctoral degree as the highest completed degree self‐assessed their competence in clinical gerontological nursing as lower than other groups. This is understandable if these participants work mainly at a management level. Considering the average competences of vocational graduates, this may be explained by the subordinate position of LPNs at the professional level [4]. Alongside the degree, continuous education does not necessarily guarantee that an LPN's job description matches their level of competence [4, 5]. This illustrates the challenges of competence allocation. LPNs have identified uncertain career paths and challenges to professional development as factors that undermine the attractiveness of the profession [4]. To ensure a sufficient and competent workforce in the future, it is important to facilitate fluent teamwork among LPNs and RNs [4], provide LPNs with sufficient opportunities for continuous education [29] and ensure sufficient competence identification.

### Study limitations

The research was carried out in one well‐being services county in Finland. The results of this study should be treated critically, as this study provides insights into the clinical gerontological nursing competence reported by LPNs working in one national area and LPNs working in remote home care, private service units, service management, or security alarm centres were excluded. More research is needed nationally and internationally to gather information that, most importantly, focuses on LPNs' competences, so that it can be reliably used and applied to other cultures and countries. The instrument used evaluates issues that may be emphasised differently in the education of LPNs compared to that of RNs. We didn't ask about the extent or duration of the continuous educations, which is also a limitation. The participants self‐assessed their competence levels, which may have affected the study results if they overestimated or underestimated their competence. Therefore, objective data is lacking. It is also possible that the study was mainly answered by highly competent LPNs. The results of the study should be viewed critically because education and experience increase awareness of what one doesn't know. So, respondents who have attended education and training may have been critical in their assessment of knowledge. This study focused on specific gerontological competences, and the other data from project work is reported in other publications. Despite the limitations, the strength of the study lies in its sample size and statistically significant results, which have been confirmed by the Benjamini‐Hochberg critical value for a false discovery rate of 0.1.

## CONCLUSION

LPNs reported having good competence in using assistive devices to support functional ability, disease‐specific nursing, wound care, pressure ulcer prevention and post‐operative wound care. However, there are differences in the self‐assessed competence of LPNs from different work units. Competence in wound care requires education. Competence assessments should specifically target those who have worked in the profession for a long time. On the other hand, mentoring should be utilised to train new graduates and employees to care for older people. The importance of continuous education should be recognised in the development of competence, job satisfaction, and retention of LPNs. The use of social media and other digital solutions in LPNs' competence development should be considered on a larger scale in the future. More research is needed with a focus on developing the competence of LPNs over 50 years of age and those in 24‐h and home care services, where clinical gerontological nursing competences were evaluated as the lowest in this study. It is important to harmonise practices, create common competence requirements and systematic competence development and management. Continuous competence assessments are useful in clarifying LPNs' career paths and thus preventing their withdrawal from the profession. The ultimate beneficiaries are older people, who receive high‐quality person‐centred care.

## AUTHOR CONTRIBUTIONS

**Suonnansalo Petra:** Conceptualization, methodology, project administration, validation, visualisation, writing—original draft; **Pramila‐Savukoski Sari:** Conceptualization, methodology, project administration, validation, visualisation, writing—original draft; **Meriläinen Merja:** Writing—review and editing; **Siira Heidi:** Writing—review and editing; **Sneck Sami:** Conceptualization, methodology, project administration; **Tohmola Anniina:** Writing—review and editing; **Karsikas Eevi:** Conceptualization, methodology, project administration, writing—review and editing; **Tuomikoski Anna‐Maria:** Conceptualization, investigation, methodology, project administration, supervision, writing—review and editing.

## FUNDING INFORMATION

The authors received no specific funding for this work.

## CONFLICT OF INTEREST STATEMENT

The authors have no conflict of interest to declare.

## ETHICS STATEMENT

This study followed the ethical principles for medical research set out in the Helsinki Declaration (World Medical Association, 2013). Permission for the research with ethical approval was granted from the board of Wellbeing Services County on 01/03/2023. Research ethics committee approval was not required since the study did not involve minors, clinical trials, or direct or indirect physical or physiological harm to the participants (Medical Research Act No. 488/1999). Informed consent was collected from each participant separately in accordance with the EU's General Data Protection Regulations (GDPR) (2016/679/GDPR).

## Data Availability

The data that support the findings of this study are available from the corresponding author upon reasonable request.
